# Supplementary material for: Metamaterial-based passive analog processor for wireless vibration sensing
Source: Commun Eng. 2024 Mar 8;3:44. doi: 10.1038/s44172-024-00190-8 (PMC10955913; doi:10.1038/s44172-024-00190-8)
Supplement: Supplementary file 2 — Supplemental material [file 44172_2024_190_MOESM2_ESM.pdf]

Supplementary Information for

Metamaterial-Based Passive Analog Processor for Wireless Vibration Sensing

*Dajun Zhang[1], Akhil Polamarasetty[2], Muhammad Osama Shahid[1], Bhuvana Krishnaswamy[1], Chu Ma[1]**

[1]Department of Electrical and Computer Engineering, University of Wisconsin–Madison, Madison, WI 53706, USA

[2]Department of Computer Sciences, University of Wisconsin–Madison, Madison, WI 53706, USA

**E-mail: chu.ma@wisc.edu*

This PDF file includes:

Supplementary Notes

1. Simulation of the devices
2. Displacement of the membrane under in-plane and out-of-plane force caused by inclination
3. Simulation of stress distribution with maximum membrane displacement

Supplementary Figures

Fig. S1. Experiment setup of vibration measurement using laser vibrometer.

Fig. S2 Displacement of the membrane under in-plane and out-of-plane force caused by inclination.

Fig. S3. Simulated stress distribution with maximum membrane displacement.

Fig. S4. Experimentally measured output vibrations of the device and their frequency spectra when the input vibration frequency is not eigenfrequency.

Fig. S5. The accuracy of the Wi-Fi based vibration detection with or without the device.

Fig. S6. Demonstration of the vibration extraction from a video using the object segmentation method.

Fig. S7. Demonstration of the vibration frequency extraction from a recorded video for the device with an eigenfrequency of 285 Hz based on the sample boundary analysis method.

Fig. S8. The influence of parameters on the device.

Supplementary Tables S1 to S2

Supplementary Table S1. The design parameters of the devices

Supplementary Algorithm Table S2. Wi-Fi sensing algorithm

Supplementary Notes

1. Simulation of the devices

We simulated the vibration properties of the devices using COMSOL Multiphysics 6.0 structural mechanics module. The 2D axisymmetric simulation is used. The Young's modulus of membrane is set as 3.89×10^{10} pa. The mass density of membrane is set as 1390 $kg \cdot m^{-3}$ and Poisson's ratio is 0.48. The other parameters used in simulations are listed in Supplementary Table S1. Those parameters are swept to observe their influence on the device's eigenfrequencies. The results are shown in Supplementary Fig. S8.

2. Displacement of the membrane under in-plane and out-of-plane force caused by inclination

We performed two simulations using the solid mechanics module in COMSOL Multiphysics. Besides the membrane (200 mm diameter, 0.15 mm thickness) and loaded mass (23.285 g), a body force (9.8 $N \cdot kg^{-1}$) with a different direction in each simulation is added to the system to simulate the rotation caused gravity direction change. The added force will offset the mass by a small distance and change the static stress distribution on the membrane. However, the results only showed a small and negligible difference between the output vibration amplitudes in the two simulations (Fig. S2).

3. Simulation of stress distribution with maximum membrane displacement

A numerical simulation is performed to check the maximum stress on the membrane. We use the Mooney-Rivlin model for the rubber thin film simulation, where the parameters C10 and C01 can be calculated approximately by these two equations:

$$E = 8 \times (C10 + C01)$$ [1]

$$C01 = 0.2 \times C10$$ [2]

where Young's Modulus E can be obtained from material technical data sheet as 0.83 MPa. In the simulation conducted by COMSOL Multiphysics 6.0 structural mechanics module, C10 is set as 0.086 MPa and C01 is set as 0.017 MPa. The diameter of the circular membrane is 200 mm and the thickness is 150 μm, the same as the membrane used in the fabricated device. We simulated two cases, one with a point load at the center, and the other with a 3 mm-diameter circular load. In both cases, the displacement at the center is 9 mm, which is the maximum displacement of the membrane with 9.5 Hz eigenfrequency reached in our experiment (shown in Fig. 2(d)). The stress distribution on the membrane is shown in Fig. S3. The peak stress on the membrane is 1.98 MPa at the center for the point-load case, and 0.037 MPa for the circular-load case, which are both much smaller than the tensile strength of the rubber film.

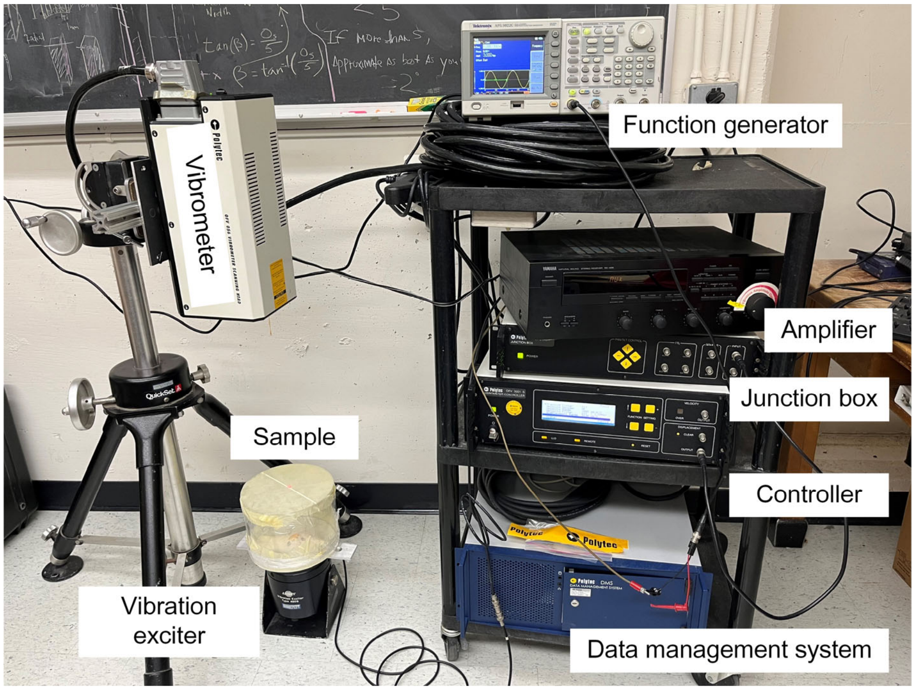

Fig. S1. Experiment setup of vibration measurement using laser vibrometer.

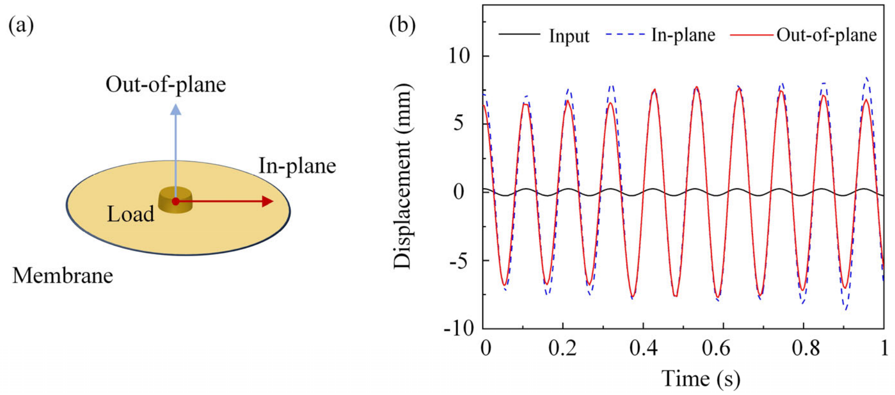

Fig. S2 Displacement of the membrane under in-plane and out-of-plane force caused by inclination. (a) Demonstration of the added in-plane (90º inclination) and out-plane force (0º inclination) in simulation and (b) Simulated input and output vibration waveforms when the input frequency is 9.5 Hz measured from the device with 9.5 Hz eigenfrequency.

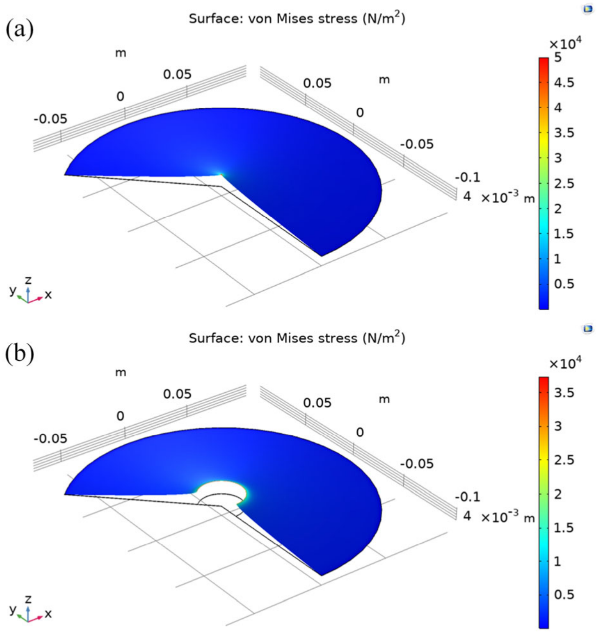

Fig. S3. Simulated stress distribution with maximum membrane displacement. The 9 mm displacement is set at the center of the membrane with (a) point load and (b) 3 mm-diameter circular load. The peak stress on the membrane is 1.98 MPa for the point-load case, and 0.037 MPa for the circular-load case.

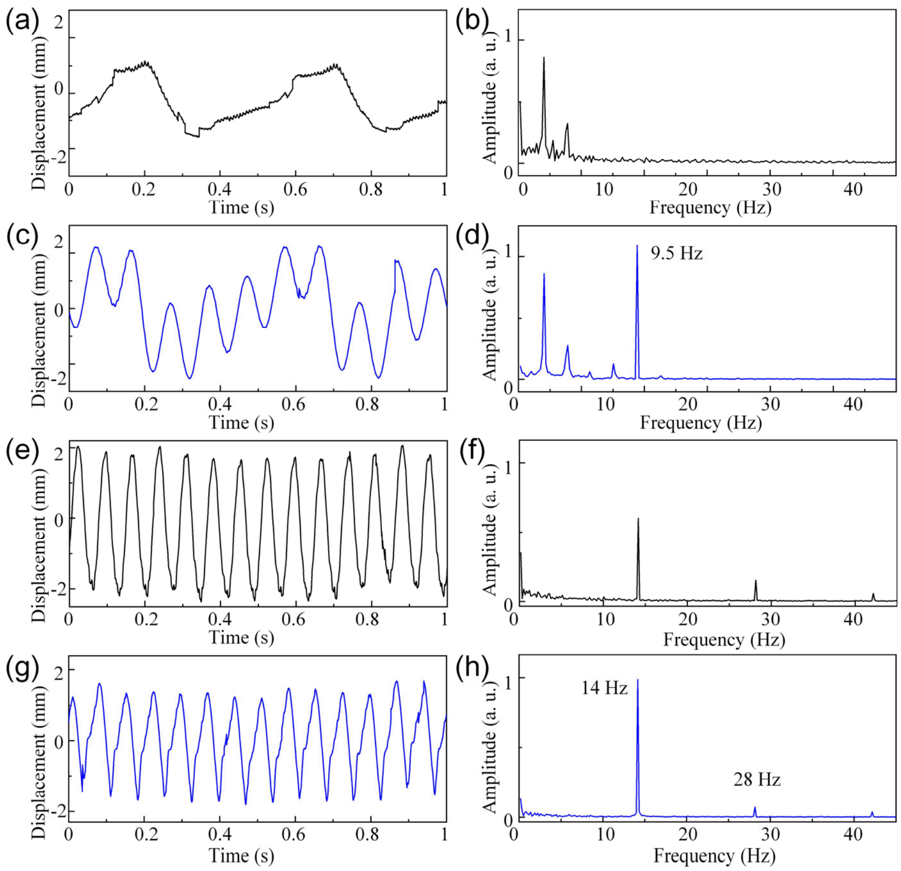

Fig. S4. Experimentally measured output vibrations of the device and their frequency spectra when the input vibration frequency is not eigenfrequency. (a-d) The input vibration (a) and output vibration (c) with a quasi 2 Hz triangle wave input. (b) and (d) are the frequency spectra of (a) and (c), respectively. (e) and (g) The input vibration (e) and output vibration (g) with a 14 Hz quasi sinusoidal wave input. (f) and (h) are the frequency spectra of (e) and (g), respectively.

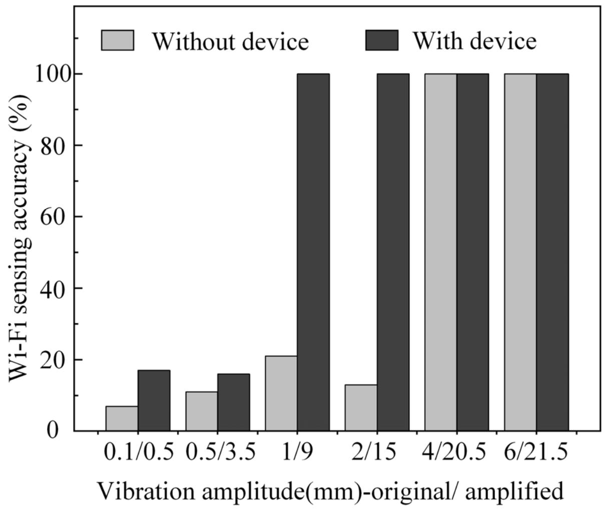

Fig. S5. The accuracy of the Wi-Fi based vibration detection with or without the device.

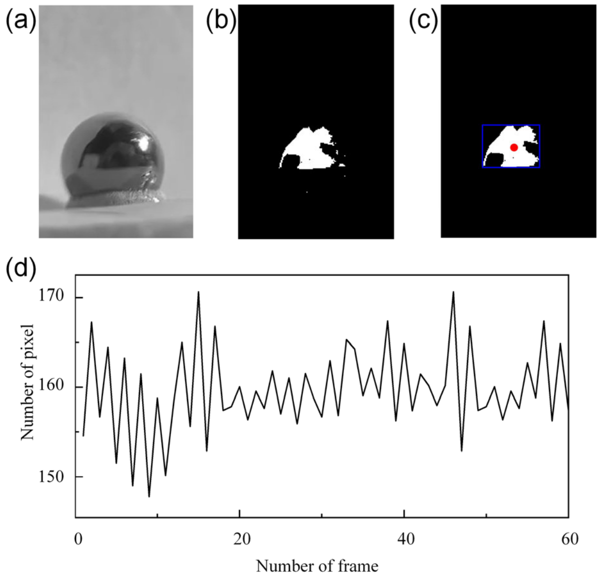

Fig. S6. Demonstration of the vibration extraction from a video using the object segmentation method. (a) A grayscale snapshot of the recorded video. (b) The black-and-white picture of (a) based on a grayscale threshold. (c) The centroid of (b) is shown as the red point. (d) The movement of the centroid as a function of time in the video, showing the vibration of the loaded mass.

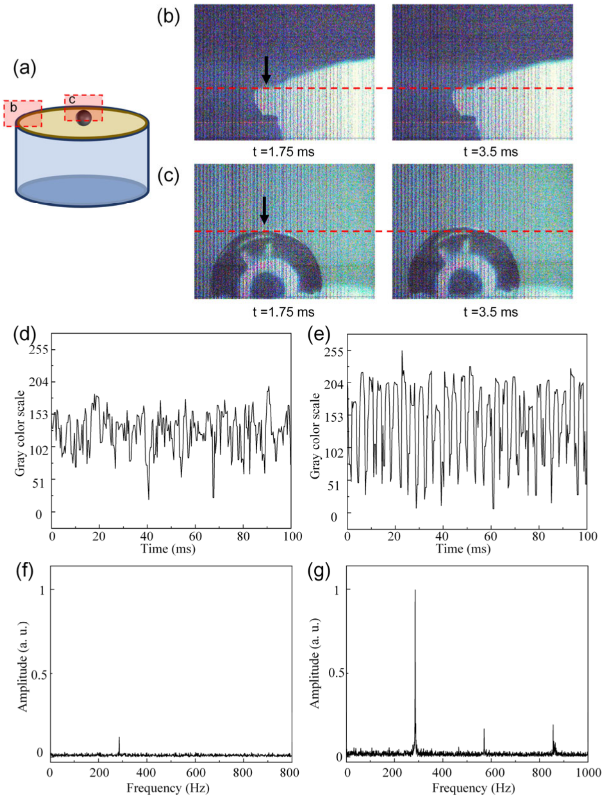

Fig. S7. Demonstration of the vibration frequency extraction from a recorded video for the device with an eigenfrequency of 285 Hz based on the sample boundary analysis method. (a) Demonstration of the recorded areas in Videos S4 (the area marked with letter b) and S5 (the area marked with letter c) for the monitoring of the outer shell and center sphere of the device. (b) The snapshots at t = 1.75 ms and t = 3.5 ms of the recorded shell movement (input vibration) from Supplementary Video S4. The movement of the outer shell is tiny and difficult to distinguish. (c) The snapshots at t = 1.75 ms and t = 3.5 ms of the recorded movement of the top sphere (input vibration) from Supplementary Video S5. (d) and (e) The grayscale value as a function of time of one point (marked with arrows in (b) and (c)) at the boundary of the outer shell (d) and the top sphere (e). (f) and (g) are the frequency spectra of (d) and (e), respectively.

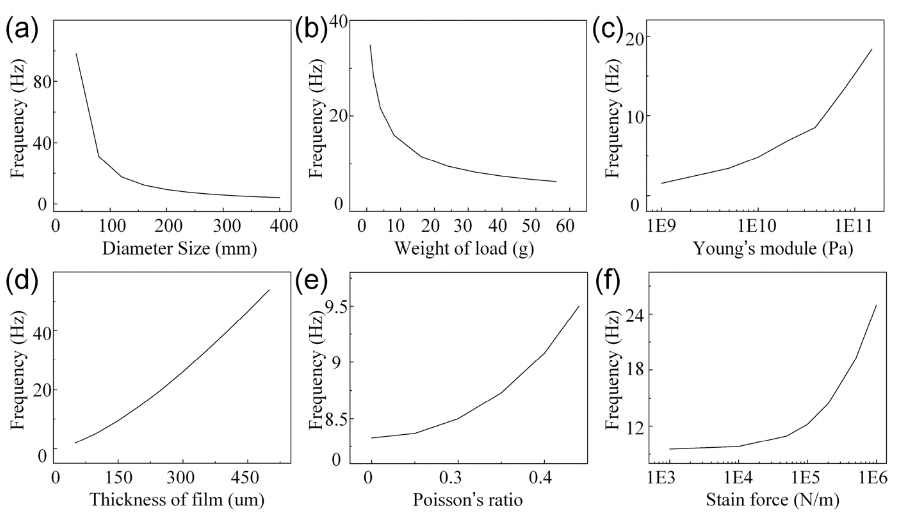

Fig. S8. The influence of parameters on the device. The first eigenfrequency of the device with the sweep of (a) membrane diameter, (b) weight of the loaded mass, (c) Young's modulus of the membrane, (d) thickness of the membrane, (e) Poisson's ratio of the membrane, and (f) static stain in the membrane. In each plot, only one parameter is swept. The initial values of all the parameters are: membrane diameter of 200 mm, loaded mass weight of 32 g, membrane Young's modulus of 38.9 GPa, membrane thickness of 150 μm, membrane Poisson's ratio of 0.48, strain stain in the membrane of 0 N.

Supplementary Table S1. The design parameters of the devices

**Device number**	**1**	**2**	**3**	**4**	**5**
Eigenfrequency	9.5 Hz	110 Hz	285 Hz	125 Hz	5 Hz
Weigth of loaded mass (g)	32	1.7	0.3	2	45
Diameter (mm)	200	20	10	20	200
Material & thickness of the film	Silicone rubber, 0.15 mm				
Membrane editing	-	-	-	fluorescent layer Coating	Loaded with a reflector
Sensing system	Wi-Fi sensing	Camera sensing	Camera sensing	Camera sensing	Wi-Fi sensing

Supplementary Algorithm Table S2. Wi-Fi sensing algorithm

Algorithm 1: WiFi sensing algorithm

Data: $CSI_{stream\text{-}1}$, $CSI_{stream\text{-}2}$, $|f_{low}, f_{high}|$

Result: $f_{1\text{-}max}, \ldots, f_{i\text{-}max}, \ldots, f_{n\text{-}max}$

start-ix = 0;

window-size = w;

finish-ix=start-ix + window-size;

phase-difference = CSI-Ratio (angle ($CSI_{stream\text{-}1}$), angle ($CSI_{stream\text{-}2}$));

While *size(phase-difference) = finish-ix* **do**

  PSD = plomb (phase – difference (start-ix, finish-ix));

  $f_{i\text{-}max}$=arg $\max_x$(PSD);

  start-ix = finish-ix;

  finish-ix = start-ix + window-size;

end

Supplementary References

[1] Bergstrom, J. S. Mechanics of solid polymers: theory and computational modeling (Chapter 5). William Andrew, 2015.

[2] Krmela1, J., Artyukhov, A., Krmelová, V., Pozovnyi, O. Determination of material parameters of rubber and composites for computational modeling based on experiment data. Journal of Physics: Conference Series, 1741, 012047 (2021).
