# Supplementary material for: Metamaterial-based passive analog processor for wireless vibration sensing
Source: Commun Eng. 2024 Mar 8;3:44. doi: 10.1038/s44172-024-00190-8 (PMC10955913; doi:10.1038/s44172-024-00190-8)
Supplement: Supplementary file 3 — Description of Additional Supplementary Files [file 44172_2024_190_MOESM3_ESM.pdf]

Description of Additional Supplementary Files

File name: Supplementary Movie 1

Description: The vibration of the device with the eigenfrequency of 9.5 Hz in a 0 degree inclination. The input vibration has a frequency of 9.5 Hz.

File name: Supplementary Movie 2

Description: The vibration of the device with the eigenfrequency of 9.5 Hz in a 90 degrees inclination. The input vibration has a frequency of 9.5 Hz.

File name: Supplementary Movie 3

Description: The video for vibrating object segmentation and moving trajectory extraction of the device with the eigenfrequency of 110 Hz. The input vibration has a frequency of 110 Hz.

File name: Supplementary Movie 4

Description: The bottom layer movement (input vibration) of the device with the eigenfrequency of 285 Hz and input frequency of 285 Hz.

File name: Supplementary Movie 5

Description: The top sphere movement (output vibration) of the device with the eigenfrequency of 285 Hz and input frequency of 285 Hz.

File name: Supplementary Movie 6

Description: The fluorescent layer movement of the device with the eigenfrequency of 125 Hz and input frequency of 125 Hz in a bright environment.

File name: Supplementary Movie 7

Description: The fluorescent layer movement of the device with the eigenfrequency of 125 Hz and input frequency of 125 Hz in a dark environment.
